# Radiological findings of pulmonary tuberculosis in indigenous patients in Dourados, MS, Brazil*

**DOI:** 10.1590/0100-3984.2014.0070

**Published:** 2015

**Authors:** Tatiana Lachi, Mauro Nakayama

**Affiliations:** 1 Master, MD, Radiologist, Hospital Regional de Mato Grosso do Sul, Auxiliary Professor at Universidade Federal de Mato Grosso do Sul (UFMS), Campo Grande, MS, Brazil.; 2 PhD, Associate Professor at Universidade Federal da Grande Dourados (UFGD), Dourados, MS, Brazil.

**Keywords:** Radiology, Tuberculosis, Chest, Radiological findings, Indigenous population

## Abstract

**Objective:**

To describe the radiological findings of pulmonary tuberculosis in indigenous patients from the city of Dourados, MS, Brazil, according to age and sex.

**Materials and Methods:**

Chest radiographic images of 81 patients with pulmonary tuberculosis, acquired in the period from 2007 to 2010, were retrospectively analyzed by two radiologists in consensus for the presence or absence of changes. The findings in abnormal radiographs were classified according to the changes observed and they were correlated to age and sex. The data were submitted to statistical analysis.

**Results:**

The individuals’ ages ranged from 1 to 97 years (mean: 36 years). Heterogeneous consolidations, nodules, pleural involvement and cavities were the most frequent imaging findings. Most patients (55/81 or 67.9%) were male, and upper lung and right lung were the most affected regions. Fibrosis, heterogeneous consolidations and involvement of the left lung apex were significantly more frequent in males (*p* < 0.05). Presence of a single type of finding at radiography was most frequent in children (*p* < 0.05).

**Conclusion:**

Based on the hypothesis that indigenous patients represent a population without genetically determined resistance to tuberculosis, the present study may enhance the knowledge about how the pulmonary form of this disease manifests in susceptible individuals.

## Abstract (pt)

**Objetivo:**

Descrever os aspectos radiológicos da tuberculose pulmonar em pacientes indígenas da cidade de Dourados, MS, Brasil, de acordo com idade e sexo.

**Materiais e Métodos:**

Radiografias de tórax de 81 pacientes com tuberculose pulmonar, realizadas de 2007 a 2010, foram analisadas retrospectivamente por dois radiologistas, em consenso quanto à presença ou ausência de alterações. Os achados em radiografias anormais foram classificados de acordo com as alterações observadas e correlacionados com idade e sexo. Os dados foram submetidos a análise estatística.

**Resultados:**

A idade dos indivíduos variou de 1 a 97 anos (idade média de 36 anos). Os achados mais frequentes foram consolidações heterogêneas, nódulos, acometimento pleural e escavações. As regiões pulmonares superiores e o pulmão direito foram mais afetados pela tuberculose e a maioria dos pacientes (55/81 ou 67,9%) era do sexo masculino. Fibrose, consolidações heterogêneas e envolvimento do ápice pulmonar esquerdo foram significativamente mais frequentes no sexo masculino (*p* < 0,05). Presença de apenas um tipo de achado radiográfico foi mais frequente em crianças (*p* < 0,05).

**Conclusão:**

Com base na hipótese de que pacientes indígenas representam pessoas ainda não geneticamente selecionadas para a resistência à tuberculose, esta pesquisa pode ampliar o conhecimento sobre como esta doença, em sua forma pulmonar, manifesta-se em indivíduos suscetíveis.

## INTRODUCTION

Tuberculosis is one of the three leading causes of death by infectious disease in adult individuals worldwide^(1)^, which represents about two million deaths and involvement of approximately eight million people around the world per year^(2)^. About 50% of the individuals who are not treated die because of the disease^(3)^. The infection by the bacillus responsible for the tuberculosis - *Mycobacterium tuberculosis* - is the most common of the human infections^(4)^ and may be found in about one third of the world population^(1,3,5)^. According to the World Health Organization, from 5% to 10% of the infected individuals develop tuberculosis along their lives^(6)^. Approximately 85% of cases of tuberculosis affect the lung parenchyma^(7)^.

In the United States of America and in Western countries, in the 1950's, the rates of infection and death by tuberculosis decreased significantly with the development of appropriate antibiotics. Since the middle of the 1980's, the acquired immunodeficiency syndrome has led to an increase in the number of new cases of tuberculosis in Europe, United States of America and particularly in Africa^(3)^. Other causes attributed to such an increase include worsening of public health services, the high correlation between the disease and poverty, and the increased resistance of bacilli to anti-tuberculosis drugs.

The Brazilian indigenous populations are particularly susceptible to tuberculosis due to different reasons including low socioeconomic conditions, difficulty in accessing health services and immunological peculiarities^(8-10)^.

Therefore, it is important that the disease is discovered early. Chest radiography plays a fundamental role in the diagnosis^(11,12)^, since it can be rapidly performed, facilitating an early diagnosis (in a screening program, it can shorten the time to diagnosis from 25 to 6 days), potentially reducing the infection transmission and onset of secondary cases. Chest radiography is the imaging method of choice for initial evaluation of the patient and also for the disease management^(4)^. The posteroanterior view is sufficient for screening, even in pediatric patients, with a positive tuberculin test^(13,14)^.

Chest radiography is even more important for the diagnosis of tuberculosis in children. The paucibacillary condition of the disease at the pediatric age requires clinical, radiological and epidemiological criteria for the diagnosis in these patients^(15)^.

The present study is aimed at describing the radiological findings of pulmonary tuberculosis in indigenous patients of Dourados, state of Mato Grosso do Sul, Brazil, distributed according to age and sex. Few studies are found in the literature about alterations in pulmonary imaging patterns caused by tuberculosis in indigenous individuals. Additionally, considering the hypothesis that such individuals represent a population that could not yet be genetically selected for resistance to the disease, the present study may enhance the knowledge about the way pulmonary tuberculosis manifests itself in susceptible individuals.

## MATERIALS AND METHODS

The present study was approved by the Committee for Ethics in Research of Universidade Federal da Grande Dourados and was developed according to the rules for use of information contained in patients records provided on items III.3.i and III.3.t of the Brazilian Resolution CNS 196/96. The term of free and informed consent was not necessary. The present study is also in accordance with the ethical standards of the World Medical Association (Declaration of Helsinki).

This is a descriptive, quantitative and retrospective study utilizing secondary data from radiographic images and information contained in records of indigenous patients treated for pulmonary tuberculosis in a hospital of the city of Dourados, state of Mato Grosso do Sul, Brazil.

### Study population

The study subjects were indigenous patients of the ethnic groups Kaiowá, Guarani and Terena, besides other indigenous patients whose ethnic origin could not be identified due lack of information. All those patients underwent treatment for pulmonary tuberculosis in the hospital in the period from 2007 to 2010. Patients included in the study were those treated for the disease and with chest radiography performed before or during the treatment, in a total of 81 patients (81 radiographs).

Most radiographic images (56/81) were acquired before the treatment and 25/81 during the treatment, as follows: 11/81, 5/81, 4/81, 2/81, 2/81 and 1/81 about five months, fifteen days, one, two, three and four months, respectively, after treatment initiation. Other group of ten patients had radiographic images acquired after the treatment (from two months to three years after their treatment) and the imaging results are described as disease sequelae in an item separately in the results section. The patients whose radiographs could not be analyzed due to unsatisfactory image quality were excluded from the study.

### Data collection instrument

The data were collected by means of forms prepared by the authors, containing information on age, sex, ethnic origin, laboratory tests results and radiographic findings of the study population. The radiographic images were analyzed as for absence or presence of findings. The abnormal radiographs were analyzed according the findings, as follows: homogeneous or heterogeneous consolidations; presence or absence of excavations, calcifications, fibrosis, atelectasis, nodules, micronodules, lymph node enlargement, or pleural involvement. Also the number and location of the affected pulmonary areas were analyzed. The radiographic images were submitted to consensual analysis by two experienced radiologist (10- and 15-year experience in the field).

### Data analysis

A descriptive statistical analysis was made and mean and standard deviation calculations were included in the study. The chi-square and exact Fisher's tests were utilized to correlate the radiological findings with age and sex. The *softwares* utilized were Excel 2007 and SPSS version 19 for a significance level = 5%.

## RESULTS

**Figure 1:**
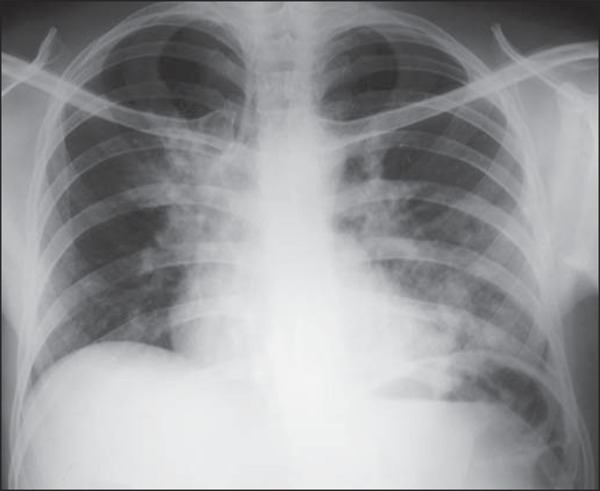
A 31-year-old man with heterogeneous and homogeneous, parahilar, bilateral pulmonary consolidations.

**Figure 2:**
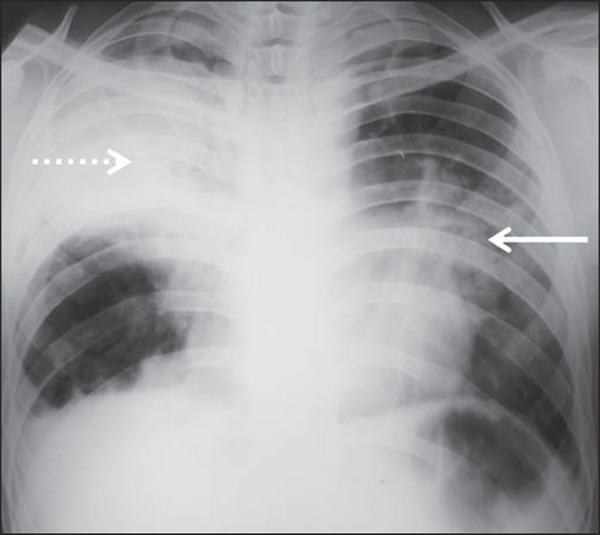
A 41-year-old man with heterogeneous (bold arrow) and homogeneous (dashed arrow) pulmonary consolidations in the left and right hemithoraces, respectively.

**Figure 3:**
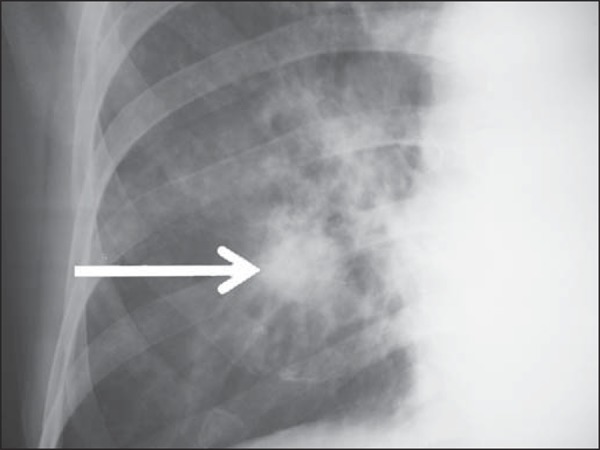
A 47-year-old man with a nodule (arrow) in the right lung.

**Figure 4:**
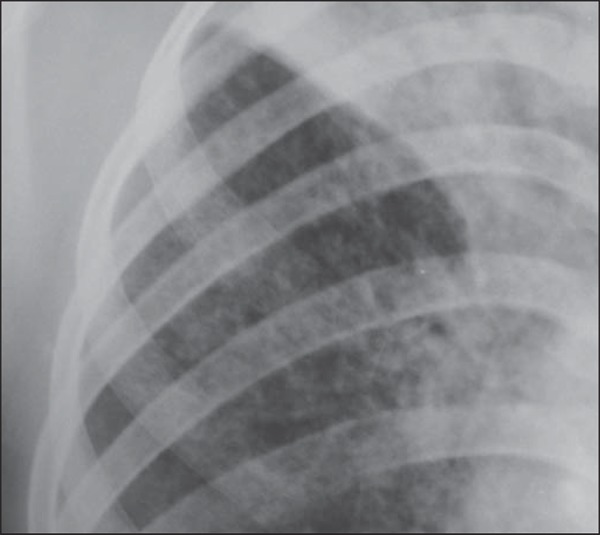
Chest radiography of a 14-year-old young girl showing the presence of pulmonary micronodules.

**Figure 5:**
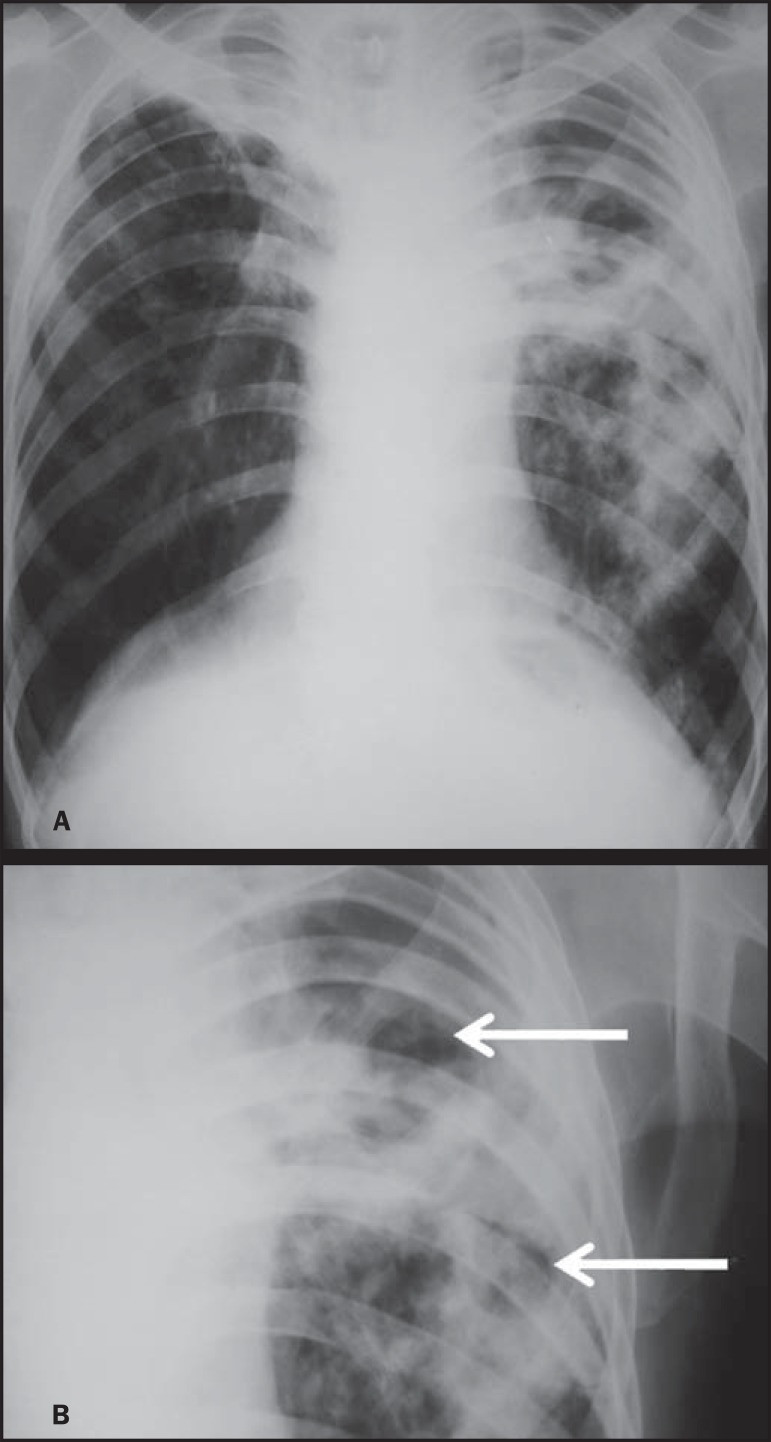
A 45-year-old man with (**A**) bilateral, superior pulmonary hilar retraction and (**B**) pulmonary excavations (arrows) without fluid level in the left hemithorax.

**Figure 6:**
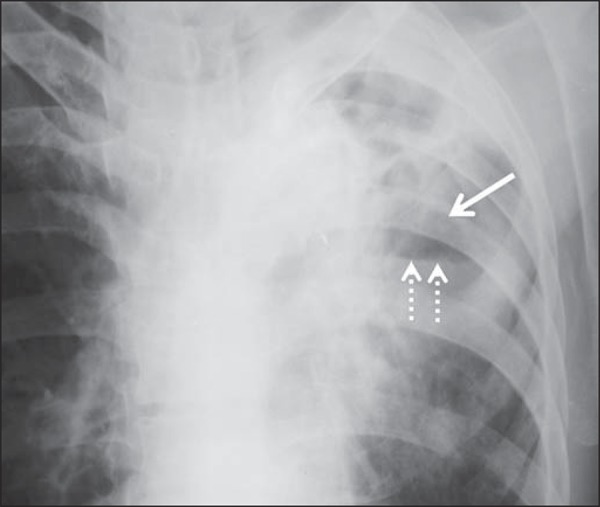
A 48-year-old man with pulmonary excavation (bold arrow) with fluid level (dashed arrows) – an uncommon finding – in the left lung.

**Figure 7:**
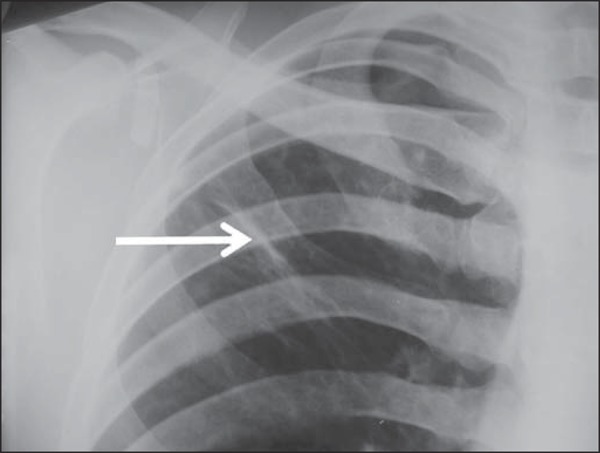
A 32-year-old man with linear density in the right lung apex (arrow). This radiographic image was acquired two years after a successful treatment for pulmonary tuberculosis.

Several imaging findings were observed in the present study. Figures 1 and 2 demonstrate the most frequent pulmonary alteration found in the study population, namely, heterogeneous consolidations. On the other hand, Figure 3 represents a nodule, while Figure 4 shows micronodules, the latter related to more severe disease. Figures 5 and 6 show bilateral superior hilar retraction and excavations, while an image representing a pulmonary tuberculosis sequela, linear density, can be seen on Figure 7.

### Distribution of patients according to age, sex, ethnic origin and radiological findings

The patients' distribution according to age and sex is shown on Table 1. Most individuals (72/81 or 88.9%) were treated on an outpatient basis. Only 9/81 (11.1%) patients were admitted to the hospital for more than 30 days.

**Table 1:** Patients' distribution according to age and sex - Dourados, MS, Brazil, 2010.

Age (years)	Number of patients	Percentage
≤ 9	7	8.6%
10-19	7	8.6%
20-29	20	24.7%
30-39	20	24.7%
40-49	11	13.6%
50-59	4	4.9%
≥ 60	12	14.8%
Sex		
Male	55	67.9%
Female	26	32.1%

The patients' mean age was 36 years (standard deviation: 21.5 years). Among the male patients, the mean age was 38.49 years (standard deviation: 20.85 years), ranging between 1 and 97 years. Among the female patients, the mean age was 30.62 years (standard deviation 22.22 years), ranging between 1 and 76 years.

The Kaiowá ethnic group represented the majority of patients, with 53/81 (65.4%) individuals, followed by the Guarani and Terena ethnic groups, with 14/81 (17.3%) and 7/81 (8.6%) individuals, respectively. Information about the ethnic origin of 7/81 (8.6%) patients was not available.

Abnormal radiographic images were found in 79/81 (97.5%) cases. Most patients (50/81 or 61.7%) presented three or more pulmonary areas affected by tuberculosis. Such a disease extent was present in 32/55 (58.2%) men and in 18/26 (69.2%) women. Only 12/81 (14.8%) patients presented only one pulmonary area affected by the disease.

Table 2 shows that the upper pulmonary areas as well as the right lung were most affected.

**Table 2:** Distribution of the affected lung areas - Dourados, MS, Brazil, 2010.

Affected lung areas	Number of patients	Percentage
Right apex	54	66.7%
Left apex	50	61.7%
Right middle third	50	61.7%
Left middle third	47	58.0%
Right lower third	39	48.1%
Left lower third	24	29.6%

The frequencies of each radiological finding are shown on Table 3. As previously mentioned, one can notice that heterogeneous consolidations were the most frequent findings in the present study.

**Table 3:** Frequency of radiological findings - Dourados, MS, Brazil, 2010.

Radiological findings	Number of patients	Percentage
Heterogeneous consolidations	69	85.2%
Nodules	38	46.9%
Pleural involvement	32	39.5%
Excavations	18	22.2%
Micronodules	13	16.0%
Fibrosis	13	16.0%
Homogeneous consolidations	12	14.8%
Lymph nodes involvement	7	8.6%
Linear densities	4	4.9%
Atelectasis	3	3.7%
Calcifications	2	2.5%

Correlation between sex and frequency of findings was observed as follows: a) fibrosis, present in 12/55 (21.8%) men and in 1/26 (3.8%) women (*p* = 0.04); b) heterogeneous consolidations, present in 51/55 (92.7%) men and in 18/26 (69.2%) women (*p* = 0.015); c) involvement of the left lung apex, present in 38/55 (69.1%) men and in 12/26 (46.2%) women (*p* = 0.047). No statistical difference was observed between male and female patients (*p* > 0.5) as regards frequency of other types of radiographic findings, likewise as regards frequency of involvement of the different pulmonary areas (except for left lung apex, as already described), frequency of abnormal radiographic images or presence of only one type of radiographic finding.

The presence of only one type of radiographic finding was observed in 20/81 (24.7%) patients and was significantly associated with the patient's age range, as follows: present in 5/7 (71.4%) children aged ≤ 9 years, in 1/7 (14.3%) adolescents between 10 and 19 years, and in 14/67 (20.9%) adult individuals aged ≥ 20 years. The frequency was higher in children as compared with adolescents (*p* = 0.031) and adults (*p* = 0.004), but no difference was observed in frequency as adolescents were compared with adults (*p* = 0.679). No statistically significant difference was observed between the three age ranges - children ≤ 9 years, adolescents between 10 and 19 years, and adults ≥ 20 years - as regards frequency of the different types of radiographic findings, frequency of involvement of different pulmonary areas, or frequency of abnormal radiographic images.

Most patients presenting with radiographic findings - 59/79 (74.7%) - had more than one type of finding, regardless the number of affected pulmonary areas.

Figures 3 to 6 show some of the radiographic findings observed in the patients of the present study. The point to be highlighted is the severity observed at the radiographic images presenting a great extent of the affected areas and severity of the findings such as presence of diffuse micronodules (Figure 4).

None of the children presented with lymph node involvement, which was found in 7/81 (8.6%) adult patients in the age range between 27 and 76 years. Similarly, atelectasis was present only in adults - 3/81 (3.7%) patients aged 35, 56 and 89 years.

In the present study, human immunodeficiency virus (HIV) serology was negative in 54/81 (66.7%) patients. In 13/81 (16.0%) and 5/81 (6.2%) individuals such a test had not been performed or was being made, respectively. No information was available about HIV serology in 9/81 (11.1%) patients. As only adults were considered, most of them were HIV-negative - 52/67 (77.6%). Most children (5/7 or 71.4%) and adolescents (4/7 or 57.1%) were not tested for HIV. Among those who were tested for HIV, 2/7 (28.6%) children and 3/7 (42.9%) adolescents had negative results.

### Pulmonary tuberculosis sequelae at radiographs

A total of 10 patients had radiographs performed at least two months after the treatment, as follows: a 33-year-old woman and 9 adult men in the age range between 24 and 81 years (mean age = 38.67 years, standard deviation 17.36 years). Human immunodeficiency virus serology was negative in 5/10 patients, and one of them did not have the test done. No information was available about the presence of the virus in 4/10 patients.

The radiograph of the woman showed heterogeneous consolidations and nodules in the apex and in the lower third of the left lung. The radiographs of the men showed heterogeneous consolidations in 5/9, linear density in 2/9, pleural involvement in 2/9, fibrosis in 1/9, nodules in1/9, excavations in1/9, and homogeneous consolidations in 1/9. The radiographic image of one of the male patients was normal.

Figure 7 shows linear density as a pulmonary tuberculosis sequela in one of the studied patients.

## DISCUSSION

The assessment of the chest by imaging methods has been object of a series of recent publications in the Brazilian radiological literature^(16-27)^. Chest radiography is an excellent imaging method in the evaluation of pulmonary tuberculosis^(4)^.

In the present study, the chest radiographic images showed a higher frequency of tuberculosis in male patients, in agreement with the literature approaching tuberculosis in general. According to the Brazilian Ministry of Health, in 2007, the incidence of tuberculosis in the country was 51/100,000 among men, and 26/100,000 among women^(28)^.

Also, the preference of the bacillus for the upper areas of the lungs in the patients of the present study is in agreement with reports in the literature for non-indigenous patients. The greater oxygen concentration in such pulmonary areas favors the bacilli development^(29)^.

Additionally, in the present study, it was observed that the right lung was preferred by the bacillus, which is not different from what is observed in the general population^(15,30-32)^.

In the present study, only 2/81 (2.5%) patients presented normal radiographic images. The high frequency of radiographic findings in the study population should be taken into consideration as one analyzes a chest radiograph of an indigenous patient with suspected pulmonary tuberculosis, as such imaging method hardly presents a normal reading in these patients. Such a result is different from other studies. Radiographic images of indigenous Suruí patients of the Amazon state, treated for pulmonary tuberculosis, were analyzed by Basta et al.^(33)^ in 2003 and 2004, and 8/22 (36.4%) of them did not present any abnormality. However, such patients were treated before all the diagnostic possibilities had been covered. In another study, Pepper et al.^(34)^ have detected 53/601 (9%) normal radiographic images among patients who presented with respiratory culture-positive for tuberculosis, referred to Nashville, Tennessee, USA. The HIV-infected patients had increased probability of presenting normal radiographic images. The probably low prevalence of HIV infection in the indigenous population included in the present study may explain the low number of normal radiographic images. However, further studies are necessary to confirm such a hypothesis.

As regards age, the different age ranges were correlated with the presence of only one type of radiological finding. As compared with adolescents and adult individuals, the children had a much higher frequency of such finding at their radiographic images, probably because they have not been reinfected with the bacillus yet.

As regards sex, it was observed that fibrosis, heterogeneous consolidation and involvement of the left lung apex were most frequently found in the male patients. A possible reason for the higher frequency of fibrosis and heterogeneous consolidations in men is the fact that they are more susceptible to the disease than the women. The reason for the difference between sexes in the involvement of the left lung apex should be object of further studies.

On the other hand, atelectasis was present only in adults, and the reasons for this should be object of specific investigations. Atelectasis is a finding resulting from compression of the trachea or of the bronchi by enlarged lymphatic ganglia. It also may be caused by endobronchial tuberculosis^(35)^.

In the present study, it was not possible to correlate radiological findings of pulmonary tuberculosis with HIV serology due to the absence of HIV-positive patients in the study population. Typically, the Brazilian indigenous peoples present a low prevalence of HIV infection^(36,37)^.

There was a great variety of radiological findings at the images, and most patients presented more than one type of visible alteration. This means that an indigenous patient in the study population with pulmonary tuberculosis presents a higher probability of having more than one type of radiographic finding, rather than only one type of radiological alteration. The types of alterations observed at the radiographic images were not different from those observed in the general population^(4)^.

Heterogeneous consolidations were the most frequent finding at the radiographic images in the present study, similarly to the findings reported by Basta et al.^(33)^. Such an imaging finding was also more frequent in the cases of pulmonary tuberculosis sequelae, but the low number of patients whose radiographic images were acquired after treatment did not allow for the analysis of the correlation between radiological findings and age and sex.

The severity of the disease in the studied indigenous patients should be highlighted. It is known that the high incidence of tuberculosis in the Brazilian indigenous population may be related to certain characteristics of those individuals, namely, difficult access to health services, inappropriate housing conditions with poor ventilation and high concentration of people in a single residence, illiteracy, high malnutrition rates, intestinal parasitic diseases, alcoholism, as well as immunological particularities^(38)^. Additionally, in the group of indigenous patients there are linguistic and cultural barriers which make the treatment more difficult^(39)^. These people live in villages, with poor conviviality with non-indigenous people. All these factors may explain the severe pulmonary involvement frequently observed in the present study, and such association should be object of further studies.

As regards limitations of the present study, it should be mentioned that some data could not be collected, considering the retrospective nature of the analysis. Few radiographic images of patients of the ethnic groups Guarani and Terena were found, precluding the correlation of findings with the different indigenous groups. Additionally, the lymph node involvement by tuberculosis can be better characterized at computed tomography as compared with radiography. However, computed tomography images were not available in the records of the patients included in the present study. The limitation of radiography in detecting lymph node involvement may explain the absence of this finding in the studied children.

## CONCLUSION

Considering that chest radiography is a rapid imaging method, it is critical to aid in the early diagnosis of pulmonary tuberculosis. The wide range of radiological findings of the disease in the patients included in the present study indicates that the understanding of such findings is essential for the agility of diagnosis, as well as for the treatment and follow-up of the disease in this population. Based on the accepted hypothesis that indigenous patients represent a population without genetically determined resistance to tuberculosis, the radiological study related to the pulmonary involvement by this disease in the indigenous population is essential to improve the diagnosis of pulmonary tuberculosis in susceptible individuals.
